# Change in Hepatitis B Surface Antibody Titers After Chemotherapy in Patients With Hematological Malignancies

**DOI:** 10.7759/cureus.51572

**Published:** 2024-01-03

**Authors:** Tülay Unver Ulusoy, Pınar Tıglıoglu, Hacer Demirköse, Murat Albayrak, İrfan Şencan

**Affiliations:** 1 Department of Infectious Diseases and Clinical Microbiology, Ankara Etlik City Hospital, Ankara, TUR; 2 Department of Infectious Diseases and Clinical Microbiology, Dışkapı Yildirim Beyazit Training and Research Hospital, University of Health Sciences, Ankara, TUR; 3 Department of Hematology, Dr. Ersin Arslan Training and Research Hospital, Gaziantep, TUR; 4 Department of Public Health, Pursaklar District Health Directorate, Ankara, TUR; 5 Department of Haematology, Ankara Etlik City Hospital, Ankara, TUR

**Keywords:** hepatitis b virus prophylaxis, hepatitis b virus reactivation, chemotherapy, hematological malignancy, anti-hbs titer

## Abstract

Background

The change in hepatitis B surface antibody (anti-HBs) titers after chemotherapy (CT) in patients with hematological malignancy, affecting factors, and its clinical implications have not been sufficiently understood. Therefore, we aim to evaluate the change in anti-HBs titers and hepatitis B virus reactivation (HBVr) after CT.

Methods

This retrospective study enrolled patients with hematological malignancies who received CT between 2013 and 2021. All patients were followed up for HBVr and a change in anti-HBs titers for one year.

Results

Overall, 192 patients were included. In total, 33.9% of the patients were anti-HBs (+) and 26% of the patients were anti-HBc (+) ± anti-HBs (+). Hepatitis B virus (HBV) prophylaxis was given to 35 (70%) of 50 Anti-HBc (+) patients. Tenofovir disoproxil fumarate and entecavir prophylaxis were initiated in 25 (71.4%) and 10 (28.6%) patients, respectively. A significant decrease was found in anti-HBs titers of all patients (p=0.017). A significant decrease was also found in anti-HBs titers of HBc IgG (+) patients and those who received four or more courses of CT (p=0.025; p=0.041). HBVr was not diagnosed in any of the patients.

Conclusion

Chemotherapeutic agents administered for hematological malignancy have serious immunosuppression effects. In these patients, anti-HBs titers may decrease or become negative one year after CT. Anti-HBs titer before CT or its change after CT may not constitute a risk for HBVr patients who received HBV prophylaxis in line with current guidelines and these recommendations.

## Introduction

Hepatitis B virus reactivation (HBVr) is one of the common causes of morbidity and mortality in patients with hematological malignancies who receive cytotoxic chemotherapy (CT) [1]. The natural progression of hepatitis B virus (HBV) infection is affected by the host's immune response to viral replication [2]. Current knowledge of the immune-mediated pathogenesis of HBV demonstrates that small quantities of HBV deoxyribonucleic acid (DNA) persist for years as episomes (ccc-DNA) in the hepatocyte nucleus, even in hosts who are clinically and biochemically disease-free.

HBVr has been regarded as a complication of immunosuppressive treatments, particularly CT [3]. CT-induced immunosuppression leads to a rapid increase in viral replications and antigen expression in hepatocytes. Between courses and/or after CT withdrawal, renewal of immune function causes rapid T cell-mediated destruction of HBV-infected hepatocytes that manifests clinically as asymptomatic self-limiting to severe hepatitis, hepatic failure, and even death [4]. Additionally, viral reactivation and the subsequent liver enzyme flare-up may impact negatively the clinical course of the hematological malignancy and prevent completion of the planned duration of cytotoxic CT. Therefore, it is recommended to screen for HBV surface antigen (HBsAg), antibody to hepatitis B core antigen (anti-HBc), and HBV DNA if anti-HBc is positive before CT and to initiate antiviral prophylaxis by risk scaling according to the CT protocols to be applied [5-7].

Guidelines on the management of HBV infection do not recommend routine testing for hepatitis B surface antibodies (anti-HBs) in patients with planned CT for hematological malignancy [1,4-6]. Anti-HBs is traditionally considered protective antibodies, which can prevent HBV infection [8]. In some previous studies, it has been reported that high anti-HBs titer may have a protective effect against HBVr or low anti-HBs titer is a risk for HBVr in patients with isolated anti-HBc (+) or resolved infection [9-13]. The change in anti-HBs titers after CT in patients with hematological malignancy, affecting factors, the duration of change, and its clinical implications have not been sufficiently understood [9]. In this study, we aimed to evaluate the change in anti-HBs titers and HBVr after CT in patients with hematological malignancies.

## Materials and methods

This retrospective study enrolled patients who were diagnosed with hematological malignancy at the Health Sciences University Dışkapı Yıldırım Beyazıt Training and Research Hospital between 2013 and 2021. Inclusion criteria were as follows: having hematological malignancy, being diagnosed by the hematology clinic, consulted the infectious diseases and clinical microbiology clinic for evaluation of HBV prophylaxis before CT, hospitalized, given CT, followed up for at least one year after CT, and over 18 years of age. Exclusion criteria were as follows: receiving immunosuppressive drugs other than CT, presence of chronic liver disease due to an etiology other than HBV, vaccinated against HBV after CT, follow-up by another institution or lost to follow-up, having incomplete demographic or laboratory data, and age below 18 years.

Patients were tested with HBsAg, Anti-HBs, and Anti-HBc before the initiation of CT. Hepatitis B markers were screened using the enzyme-linked immunosorbent assay (ELISA) method (ETIMAX 3000, DiaSorin, Saluggia, Italy). Follow-up anti-HBs titer was evaluated after the first year of CT. Anti-HBs levels above 10 IU/mL were considered positive (+), and those below were considered negative (-). As per current guideline recommendations, antiviral prophylaxis was initiated in all anti-HBc (+) high-risk patients with HBVr (receiving rituximab-based CT) and some moderate-risk patients according to the physician's decision. Antiviral prophylaxis was initiated one to three weeks before CT and continued for 6-18 months after the end of the last course of CT, depending on the type of CT [2,4-6]. All patients were followed up for HBVr. Reactivation was described as the detectable HBV DNA and/or HBsAg seroreversion in blood tests during the follow-up. The data of the patients were collected retrospectively from their medical records. This study was approved by the Ethical Committee of Health Sciences University Dışkapi Yıldırım Beyazıt Training and Research Hospital (decision number: 113/19, decision date: 14.06.2021).

Organization and analysis of the research data

The following variables were examined in the study: age, gender, presence of comorbidities (such as diabetes and hypertension), receiving HBV prophylaxis, patient outcome one year after CT (exitus, survived), hematological diagnosis (Hodgkin lymphoma, non-Hodgkin lymphoma, chronic lymphocytic leukemia, acute myeloid leukemia, acute lymphoblastic leukemia, acute promyelocytic leukemia, myelodysplastic syndrome [MDS]), CT protocol, number of CT courses and hepatitis markers (anti-HBs, anti-HBc), aspartate aminotransferase (AST) and ALT values, and hepatobiliary ultrasonography report. Age groups were categorized according to the median age. Hematological diagnoses were also categorized into two groups: lymphoproliferative diseases and acute leukemia and MDS.

The SPSS (Statistical Package for the Social Sciences) Version 29 (IBM Corp., Armonk, NY) was used for the statistical analysis of the data. Categorical variables were presented as numbers and percentages, and continuous variables were presented as mean ± standard deviation and median (min-max). The variables that refer to the times and their distribution were expressed as the median and as 25th and 75th percentiles. As a statistical method, the McNemar test was used for categorical variables, and the Wilcoxon test was used for non-parametric variables. The statistical significance value was considered as p<0.05.

## Results

Overall, 192 patients with the diagnosis of hematological malignancies were included in this study. In total, 33.9% of the patients were positive for anti-HBs, and 26% were positive for anti-HBc. HBsAg and HBV DNA PCR (polymerase chain reaction) were negative in all patients. Demographic and clinical characteristics and laboratory results of the patients before CT are presented in Table 1.

**Table 1:** Demographic characteristics, clinical findings, and HBV markers of the patients COI, cutoff index value; HBc, antibody to hepatitis B core antigen; HBs, hepatitis B surface antibody; HBV, hepatitis B virus

	n=192	Percentage (%)
Age		
Mean ± SD	53.2 ± 15.9	
Gender		
Female	82	42.7
Male	110	57.3
Comorbidity		
Yes	82	42.7
No	110	57.3
Diabetes mellitus		
Yes	33	17.2
No	159	82.8
Hypertension		
Yes	49	25.5
No	143	74.5
HBV prophylaxis		
Yes	35	18.2
No	157	81.8
Anti-HBs, IU/mL		
Positive (≥10)	65	33.9
Negative (<10)	127	66.1
Anti-HBc, COI		
Positive (≥1)	50	26
Negative (<1)	142	74
Anti-HBc (+), anti-HBs (+)	31	16.1
Anti-HBc (+), anti-HBs (-)	19	9.8
Anti-HBc (-), anti-HBs (+)	34	17.7
Patient outcome		
Exitus	43	22.4
Survived	149	77.6

Hematological diagnoses of the patients and the CT protocols are shown in Table 2.

**Table 2:** Evaluation of hematological diagnoses and CT protocols of the patients CT, chemotherapy

	n	Percentage (%)	
Disease group		
Lymphoproliferative diseases	117	60.9	
Acute leukemia and myelodysplastic syndrome	75	39.1	
Hematological diagnosis			
Hodgkin lymphoma	38	19.8	
Non-Hodgkin lymphoma	54	28.1	
Acute myeloid leukemia	48	25	
Chronic lymphocytic leukemia	25	13	
Acute lymphoblastic leukemia	8	4.2	
Acute promyelocytic leukemia	1	0.5	
Myelodysplastic syndrome	18	9.4	
CT protocol			
Rituximab-based	77	40.1	
Alkylating agent and/or anthracycline-based	40	20.8	
Demethylating agent-based	24	12.5	
High-dose CT	49	25.5	
Other CT regimens	2	1	
Number of CT courses			
1	49	25.5	
2	4	2.1	
3	1	0.5	
4	7	3.6	
5	1	0.5	
6 and above	130	67.9	

Table 3 lists the laboratory results of patients receiving HBV prophylaxis before CT. HBV prophylaxis was given to 35 (70%) of 50 anti-HBc (+) patients. HBV prophylaxis was given to all anti-HBc positive patients receiving rituximab-based CT. Tenofovir disoproxil fumarate and entecavir prophylaxis were initiated in 25 (71.4%) and 10 (28.6%) patients, respectively. HBVr was not diagnosed in any of the patients.

**Table 3:** Laboratory results of patients who received HBV prophylaxis AST, aspartate aminotransferase; ALT, alanine aminotransferase; COI, cutoff index value; HBc, antibody to hepatitis B core antigen; HBs, hepatitis B surface antibody; HBV, hepatitis B virus

	n	Percentage (%)
AST (IU/L); reference range: 0-40		
Mean ± SD	21.31± 11.24	
ALT (IU/L); reference range: 0-41		
Mean ± SD	20.94± 10.98	
Anti-HBs, IU/mL		
Positive (≥10)	22	62.9
Negative (<10)	13	37.1
Anti-HBc, COI		
Negative (≥1)	0	0
Positive (<1)	35	100
Hepatobiliary ultrasonography		
Hepatic contours are regular, parenchymal echo is homogeneous	26	74.2
Hepatic contours are regular, parenchymal echo is homogeneous, grade 1 hepatosteatosis	6	17.1
Hepatic contours are regular, parenchymal echo is homogeneous, grade 2 hepatosteatosis	3	8.6

The change in anti-HBs titers of the patients before and after CT were evaluated (Table 4). A significant decrease was found in anti-HBs titers of all patients (p=0.017). A significant decrease was also found in anti-HBs titers of HBc IgG (+) patients and those who received four or more courses of CT (p=0.025; p=0.041). Among patients undergoing four courses or more of CT, 55.1% received rituximab-based CT.

**Table 4:** Comparison of anti-HBs titers of patients before and after CT COI, cutoff index value; CT, chemotherapy; HBc, antibody to hepatitis B core antigen; HBs, hepatitis B surface antibody; MDS, myelodysplastic syndrome

Subgroups	n	Pretreatment median (IQR: 25-75), lU/mL	Posttreatment median (IQR 25-75), lU/mL	p-value
All patients	192	0 (0-43.5)	0 (0-31.5)	0.017
Female	82	0 (0-36.25)	0 (0-23.25)	0.056
Male	110	0 (0-54.92)	0 (0-34.5)	0.135
<57 years of age	97	0 (0-24)	0 (0-22.5)	0.121
≥57 years of age	95	0 (0-52)	0 (0-43)	0.076
Anti-HBc positivity (COI ≥ 1)	50	36.5 (0-214.75)	23.5 (0-166)	0.025
Lymphoproliferative diseases	117	0 (0-29)	0 (0-16.5)	0.092
Acute leukemia and MDS	75	0 (0-60)	0 (0-45)	0.103
Rituximab-based CT	77	0 (0-46.07)	0 (0-22)	0.420
Alkylating agent and/or anthracycline-based CT	40	0 (0-21.75)	0 (0-16.75)	0.161
Demethylating agent-based CT	24	0 (0-84)	0 (0-74.5)	0.237
High-dose CT regimens	49	0 (0-40)	0 (0-33)	0.230
Receiving four courses or more in CT	138	0 (0-49.28)	0 (0-26.75)	0.041

Anti-HBs titers of the patients were categorized and compared before and after CT; there was a significant difference (p=0.030) (Table 5). Three people with anti-HBs titer between 10 and 100 IU/L before CT had anti-HBs titers <10 IU/L after CT. One of the three patients received rituximab-based CT, one patient received anthracycline-based CT, and one patient received demethylating agent-based CT. Anti-HBs titer was 10-100 IU/L after CT in four patients with anti-HBs titer above 100 IU/L before CT. Three of these patients received rituximab-based CT and one received demethylating agent-based CT.

**Table 5:** Comparison of anti-HBs titers before and after CT according to the categories p: McNemar test

Anti-HBs titers before CT	Anti-HBs titers after CT			
	<10	10-100	>100	Total
<10	127	0	0	127
10-100	3	27	0	30
>100	0	4	31	35
Total	130	31	31	192
p-value	0.030			

## Discussion

In this study, patients with various hematological malignancies who received several types of CT were investigated, and it was observed that anti-HBs titers decreased after CT. On the other hand, none of the patients with decreased anti-HBs titer or anti-HBs (-) patients for whom HBV prophylaxis was indicated developed HBVr.

Chemotherapeutics administered in the treatment of hematological malignancies reduce cancer-related deaths and prolong life expectancy. However, HBVr is a significant risk in this group of patients [1]. The administration of immunosuppressive CT affects the balance between the host’s immune system and viral replication, thus suppressing normal immunological responses and affecting cellular and humoral immune responses [14]. The underlying mechanism in the decrease of anti-HBs titer after CT is probably the destruction of antibodies producing B lymphocytes by CT. After exposure to HBV, CD4+ T cells trigger activation of both cytotoxic T cells and B cells [15].

Multiple factors such as HBsAg positivity, HBV DNA levels, anti-HBc positivity, the effect of the patient's malignancy on the immune system, and the type of CT underlie the presence of HBVr [5,6]. The change in anti-HBS titer in patients with hematological malignancies receiving CT and its relation to HBVr is under study. In some previous studies, anti-HBs titer decreases after CT in patients with hematological malignancies [12,16-19]. In some studies, it was determined that anti-HBs became negative after CT in anti-HBs (+) patients [12,17,18]. Studies suggested that pretreatment low-level positivity of anti-HBs is a risk factor for becoming negative for anti-HBs, particularly in patients with hematological malignancies who have undergone immunosuppressive anticancer therapy [10,17,20,21]. Consistent with the literature, in this study, a significant decrease in anti-HBs titer was detected after CT, and anti-HBs values of three patients became negative while the anti-HBs value was positive. In a study, patients with hematological malignancy receiving CT were examined; anti-HBs did not become negative in the group with anti-HBs>100, while anti-HBs negativity was reported in 8 of 19 patients with anti-HBs<100 [19]. In addition, in another study including patients with various hematological malignancies who received CT, it was reported that 12% of patients with low anti-HBs levels before CT became anti-HBs negative after CT [18]. In this study, while anti-HBs did not become negative in the group with anti-HBS>100, the results of all three people who became anti-HBs (-) were in the range of 10 of 100. These results suggested that high anti-HBs titer before CT may be protective against anti-HBs negativity after CT.

Rituximab is a chimeric monoclonal antibody against the B-cell surface antigen CD20 that is used in different B-cell lymphoid malignancies and various nonmalignant immune-mediated diseases [22]. Of great clinical importance, severe and even fatal HBVr has been defined in patients undergoing rituximab-containing CT [23]. In this study, 40.1% of patients received rituximab-based CT, and there were no patients with HBVr. This may be related to the fact that antiviral prophylaxis was initiated in all anti-HBc (+) patients who received rituximab-based CT. In previous studies, anti-HBs decrease or Anti-HBs negativity has been reported after CT in patients receiving rituximab [12,17,19]. In this study, no significant decrease in anti-HBs titer was analyzed in patients receiving rituximab-based CT. However, anti-HBs titers were significantly lower in patients who received four courses or more of CT, and 55% of these patients received rituximab-based CT. In addition, one of the three patients who were anti-HBs (-) after CT and three of the four patients whose anti-HBs titer was >100 before CT and decreased below 100 after CT received rituximab treatment.

Prophylactic nucleoside analog therapy has been shown to reduce the incidence of HBVr in HBsAg (+) or HBsAg (-) and anti-HBc (+) patients and is recommended in current treatment guidelines [1,5,6]. Randomized trials have reported that prophylactic antiviral therapy is more effective than pre-emptive strategies [24]. Studies have reported that the prophylactic use of antiviral agents before CT in these patients may reduce levels of reactivation-related morbidity and mortality [7,25]. The risk of reactivation depends on the HBV DNA level, the type of CT given, and the timely administration of antiviral prophylaxis with effective agents [26]. The ideal oral antiviral agents that can be used for prophylaxis are entecavir and tenofovir compared with the other nucleus (t)ide analogs, such as lamivudine, adefovir, and telbivudine [1,26,27]. In this study, tenofovir or entecavir prophylaxis was given to 70% of anti-HBc (+) patients one to three weeks before CT. The non-detection of HBVr may be because prophylaxis with effective agents was initiated at the optimum time following the recommendations of current guidelines in high-risk patients.

Limitations

This is a single-center study, and there are some limitations due to its retrospective design. In addition, there are some limitations due to its retrospective design. Hepatitis markers and liver enzyme tests could not be followed up at the same intervals in all patients after CT. Anti-HBs titers have not been compared with a control group not receiving CT. In addition, serial anti-HBs monitoring could not be performed in patients.

## Conclusions

Chemotherapeutic agents administered for hematological malignancy have serious immunosuppression effects. In these patients, anti-HBs titers may decrease or become negative one year after CT. Anti-HBs titer before CT or its change after CT may not constitute a risk for HBVr patients who received HBV prophylaxis in line with current guidelines and these recommendations.
